# Philadelphia Medical Society

**Published:** 1841-12-04

**Authors:** 


### PROCEEDINGS OF SOCIETIES.

### PHILADELPHIA MEDICAL SOCIETY.

*Session of Nov.* 27. Dr. Warrington read an interesting lecture on *diseases within the female pelvis producing symptoms analogous to those of displacement of the uterus.* The lecturer, after a full statement of the rational signs of prolapsus of the uterus, as laid down by authors, proceeded to comment upon the various modes in which those signs were grouped together in diseases within the pelvis in such a manner as fatally to confuse the diagnosis.

Among the affections sometimes simulating procidentia or prolapsus in many of their rational signs, were mentioned retrovertion, antiversion, and even neuralgia of the neck and body of the uterus, fibrous and polypous tumours of the pelvis, chronic metritis, spasm of the urethra, ulcerations within the vagina, lumbar and other spinal irritations, duplicature or invagination of the vagina, stricture of the rectum, &c.

The lecturer appeared to desire from the members further light, by which some settled diagnostic signs of an affection that at present seems, with the public and many professional men, to furnish an universal patronymic for all the diseases of the female pelvis about which there is found to hang some degree of mystery.

In the debate, Dr. Reynell Coates repudiated the idea that the rational signs of prolapsus as laid down by the authorities, (using the term generically in its widest sense) were in any degree necessarily characteristic of the malposition of the uterus, which latter are less important in themselves than they are usually esteemed, in cases in which the uterus does not descend so far as to present at the orifice of the vagina, or project beyond it. The various peculiar discharges, the anal and vesical symptoms, the dragging and bearing-down sensations, the lumbar uneasiness, and the sympathetic pains felt in the hypochondriac region, were all symptomatic in various complaints essentially distinct in character from malposition of the uterus, and might occur in their totality without that relaxation of the subvaginal cellulur tissue,—particularly at the upper part or the posterior face of the vagina,—which is the principal mechanical cause that permits the descent of the uterus under abdominal pressure. Among the causes of many of the broadest combinations of these *so styled* rational symptoms, he thought it admissible to rank a condition of the neck of the uterus, sometimes attended by superficial ulcerations of the part, which he believed to be of much more common occurrence than was usually supposed, especially in the class of cases in which there is a strong tendency to chronic hysterical convulsions. He admitted that prolapsus uteri very frequently occasioned a dragging and bearing-down sensation, which, however, was a necessary consequence of some forms of piles, certain pelvic tumours, vesical irritations, and even undue habitual tonicity of the abdominal muscles—not a very rare effect of long continued costiveness. He stated that the pressure of the neck of the uterus upon the perineum, in moderate cases of procidentia, sometimes produced and always very seriously exacerbated irritation of the part, and gave rise to complicated symptoms; but insisted that these were signs of the irritation and not the procidentia, and were very often present in the absence of the cause to which they are usually attributed. Procidentia, he remarked, was often paroxysmal and temporary, from general relaxation, and frequently recovered spontaneously from the improvement of health and mental exhilaration; and, that it often existed for a long time without producing any materially uncomfortable consequences. "The only *rational* sign of a prolapsus uteri, is *a falling of the womb!*" And whenever the practitioner has reasonable grounds for conviction that there exists important disease within the female pelvis, he is bound to make an examination per vaginam, by which means the displacement, if existing, is immediately discovered. The physician can then judge, with tolerable certainty, how far the displacement is the cause, and to what degree it is the consequence of the other diseased affections to which what are called the rational signs are due.

Dr. Caspar Morris condemned very decidedly, on the score of morals and feminine delicacy, the extent to which examinations per vaginam are carried of late, especially in young girls. He believed it a serious matter, and a growing evil. The public had been impressed too strongly with the idea of their importance; and when any nervous and other symptoms were capable of being referred to the pelvis, even the mothers, of late years, are disposed to urge the painful and very disagreeable office upon the practitioner, in many cases where he deems it not only unnecessary, but highly improper.

He had hoped that the debate would have elicited some suggestions for the treatment of that class of *seemingly* mere nervous affections in young girls in which no structural disease or mal-position of parts whatever can be discovered, yet which are attended with so many of the rational signs of displacement of the uterus, as to give rise to frequent, painful, and distressing examinations. He spoke of the ill effects of low diet and confinement upon cases of this character, and the too frequent resort to such measures by practitioners of distinction. This remark was additionally impressed by Drs. Parrish and R. Coates, the latter advocating the adoption of exercise by means of properly regulated calisthenic amusements in a cool room, coupled with a generous but simple diet, with a view to develope the muscular system of those who are unable to enjoy the open air with freedom. The tonicity thus gained, he stated, not only removed the nervous symptoms in many cases, but even restored the uterus to its proper elevation in some not very trifling cases of procidentia, without the aid of any mechanical treatment.

